# Cell-specific secretory granule sorting mechanisms: the role of MAGEL2 and retromer in hypothalamic regulated secretion

**DOI:** 10.3389/fcell.2023.1243038

**Published:** 2023-09-18

**Authors:** Denis Štepihar, Rebecca R. Florke Gee, Maria Camila Hoyos Sanchez, Klementina Fon Tacer

**Affiliations:** ^1^ School of Veterinary Medicine, Texas Tech University, Amarillo, TX, United States; ^2^ Texas Center for Comparative Cancer Research (TC3R), Amarillo, TX, United States; ^3^ Biotechnical Faculty, University of Ljubljana, Ljubljana, Slovenia

**Keywords:** secretory granule, MAGEL2, anterograde and retrograde protein sorting, retromer, neuroendocrine cells, WASH complex, Prader-Willi and Schaaf-Yang syndromes, hormones and neuropeptides

## Abstract

Intracellular protein trafficking and sorting are extremely arduous in endocrine and neuroendocrine cells, which synthesize and secrete on-demand substantial quantities of proteins. To ensure that neuroendocrine secretion operates correctly, each step in the secretion pathways is tightly regulated and coordinated both spatially and temporally. At the *trans*-Golgi network (TGN), intrinsic structural features of proteins and several sorting mechanisms and distinct signals direct newly synthesized proteins into proper membrane vesicles that enter either constitutive or regulated secretion pathways. Furthermore, this anterograde transport is counterbalanced by retrograde transport, which not only maintains membrane homeostasis but also recycles various proteins that function in the sorting of secretory cargo, formation of transport intermediates, or retrieval of resident proteins of secretory organelles. The retromer complex recycles proteins from the endocytic pathway back to the plasma membrane or TGN and was recently identified as a critical player in regulated secretion in the hypothalamus. Furthermore, melanoma antigen protein L2 (MAGEL2) was discovered to act as a tissue-specific regulator of the retromer-dependent endosomal protein recycling pathway and, by doing so, ensures proper secretory granule formation and maturation. MAGEL2 is a mammalian-specific and maternally imprinted gene implicated in Prader-Willi and Schaaf-Yang neurodevelopmental syndromes. In this review, we will briefly discuss the current understanding of the regulated secretion pathway, encompassing anterograde and retrograde traffic. Although our understanding of the retrograde trafficking and sorting in regulated secretion is not yet complete, we will review recent insights into the molecular role of MAGEL2 in hypothalamic neuroendocrine secretion and how its dysregulation contributes to the symptoms of Prader-Willi and Schaaf-Yang patients. Given that the activation of many secreted proteins occurs after they enter secretory granules, modulation of the sorting efficiency in a tissue-specific manner may represent an evolutionary adaptation to environmental cues.

## 1 Introduction

**FIGURE 1:**
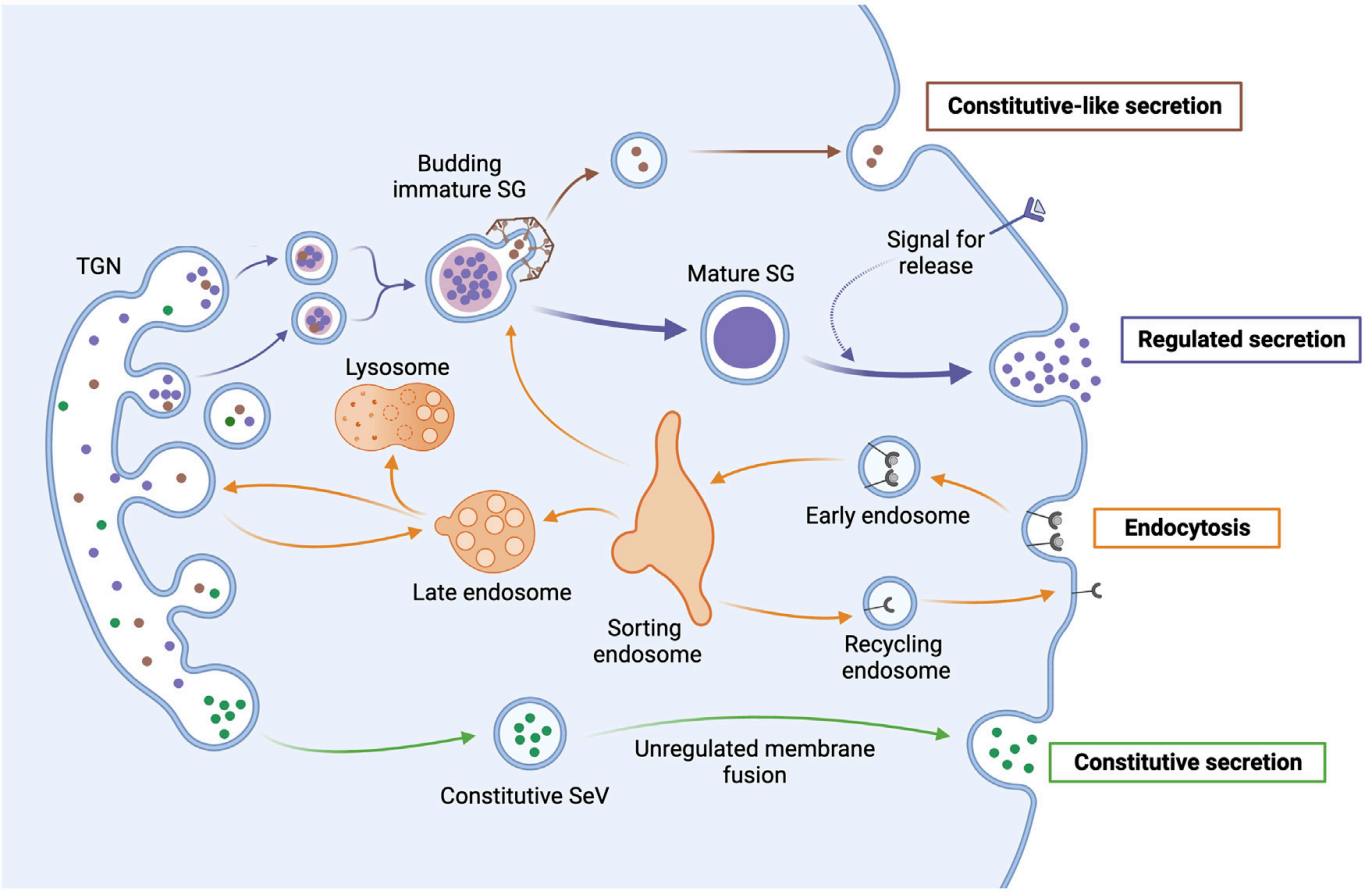
Anterograde and retrograde transport pathways in secretory cells. After protein synthesis in the ER, secretory proteins are sorted in the TGN through import signals, post-translational modifications, and other oligomeric associations. In the regulated secretion pathway, SGs go through a maturation process that includes fusion with other immature SGs and condensation of cargo proteins, as well as the removal of excess membrane and missorted cargo through the budding of clathrin-coated constitutive-like vesicles that may be secreted. Mature SGs accumulate near the plasma membrane until receiving a signal to undergo exocytosis and release their contents. In contrast, secretory vesicles (SeVs) in the constitutive secretion pathway continuously release their contents through unregulated membrane fusion. In retrograde transport, endocytosed material (e.g., receptors) are brought to a sorting endosome that directs endosomal material either back to the membrane in a recycling endosome, to immature SGs, to the lysosome for degradation, or to the TGN.

Constitutive secretion or exocytosis occurs in all cell types and predominantly facilitates housekeeping functions, including protein insertion into the plasma membrane or secretion of extracellular matrix components, growth hormones, and plasma proteins. In contrast, regulated secretion facilitates the specialized function of excitable cells (i.e., neurons and endocrine and neuroendocrine cells) which is to synthesize, store, and secrete on-demand hormones, neuropeptides, and neurotransmitters. The fundamental pathway and the basic machinery for regulated and constitutive secretion are similar, but their regulation and sorting mechanisms differ (Palade, 1975; Gerber and Sudhof, 2002). Through a series of membrane-trafficking steps, secretory proteins are synthesized in the endoplasmic reticulum (ER) and transported in membrane vesicles via the Golgi network to the plasma membrane (Palade, 1975). Unlike the continuous release of secretory molecules in the constitutive pathway, neuropeptides and hormones in the regulated secretion pathway are accumulated and stored in secretory vesicles, referred to as secretory granules (SGs), until cells receive a signal for their release through fusion with the plasma membrane (Figure 1) (Kelly, 1985). Some secreted small molecules, like neurotransmitters, are synthesized in the cytosol and taken up into synaptic vesicles (SVs) just before exocytosis (Liu and Edwards, 1997; Kogel and Gerdes, 2010). While some neurons contain only SVs or SGs, hypothalamic neurons can contain both at the same time (Thureson-Klein, 1983; Burgoyne and Morgan, 2003). Since the nomenclature and abbreviation of vesicles in constitutive and regulated secretion are sometimes confusing, we will use the terms secretory vesicles (SeVs) for organelles in the constitutive secretion pathway and secretory granules and synaptic vesicles for those in the regulated secretion pathway.

Although the two principal pathways of regulated secretion share many components, they differ in the size of the vesicles [i.e., SGs are bigger (>100 nm in radius) than SVs (<25 nm)], mechanisms by which secretory vesicles are filled with secretory molecules, and how the vesicles recycle after exocytosis for a new round of secretion (Gerber and Sudhof, 2002). In Figure 1, we schematically depict the major pathways of anterograde and retrograde trafficking and use only SGs to represent regulated secretion as SVs are beyond the scope of this review. We will focus on the regulation and sorting of cargo and resident proteins of SGs and discuss the sorting mechanisms that direct cargo and resident proteins in the anterograde transport to SGs. Furthermore, we will discuss new insights into the regulation of retrograde trafficking and how it contributes to secretion in the hypothalamus. Recently, ubiquitination-mediated regulation of retromer and F-actin nucleation was found to be critical for the recycling of resident SG proteins and the neuroendocrine function of the hypothalamus. Ubiquitination is governed by Prader-Willi associated protein MAGEL2 in conjunction with E3 ubiquitin ligase TRIM27 and deubiquitinating enzyme USP7 (Hao et al., 2013; Hao et al., 2015; Chen et al., 2020).

## 2 Brief overview of the biogenesis, maturation, and anterograde transport of secretory granules

Regulated secretion of hormones and neuropeptides is a multistep, tightly regulated process, involving protein synthesis in the ER, protein sorting and packing into SGs at the *trans*-Golgi network (TGN), SG maturation during vesicle transport from the TGN to the plasma membrane, SG storage and accumulation near the plasma membrane, and ultimately, exocytosis to release SG cargo in response to a physiological stimulus (Figure 1). For more details on these steps beyond our summary below, please refer to the following: (Tooze, 1998; Chieregatti and Meldolesi, 2005; Kim et al., 2006; Wickner and Schekman, 2008; Kogel and Gerdes, 2010; Tanguy et al., 2016; Ma et al., 2021).

In a process similar to viral budding, nascent SGs start forming from the TGN by protein accumulation that leads to GTP-dependent membrane deformation (Tooze, 1998). Cholesterol facilitates membrane bending and SG scission by promoting negative membrane curvature and recruiting proteins, like the ubiquitously expressed mechano-GTPase dynamin-2 (Wang et al., 2000; Kim et al., 2006; Gonzalez-Jamett et al., 2013; Bhave et al., 2020). After leaving the TGN, the nascent or immature SGs (ISGs) undergo maturation while transported in a microtubule-dependent manner toward the plasma membrane and the F-actin-rich cell periphery (Figure 1) (Howell and Tyhurst, 1982; Rudolf et al., 2001; Ponnambalam and Baldwin, 2003).

**FIGURE 2:**
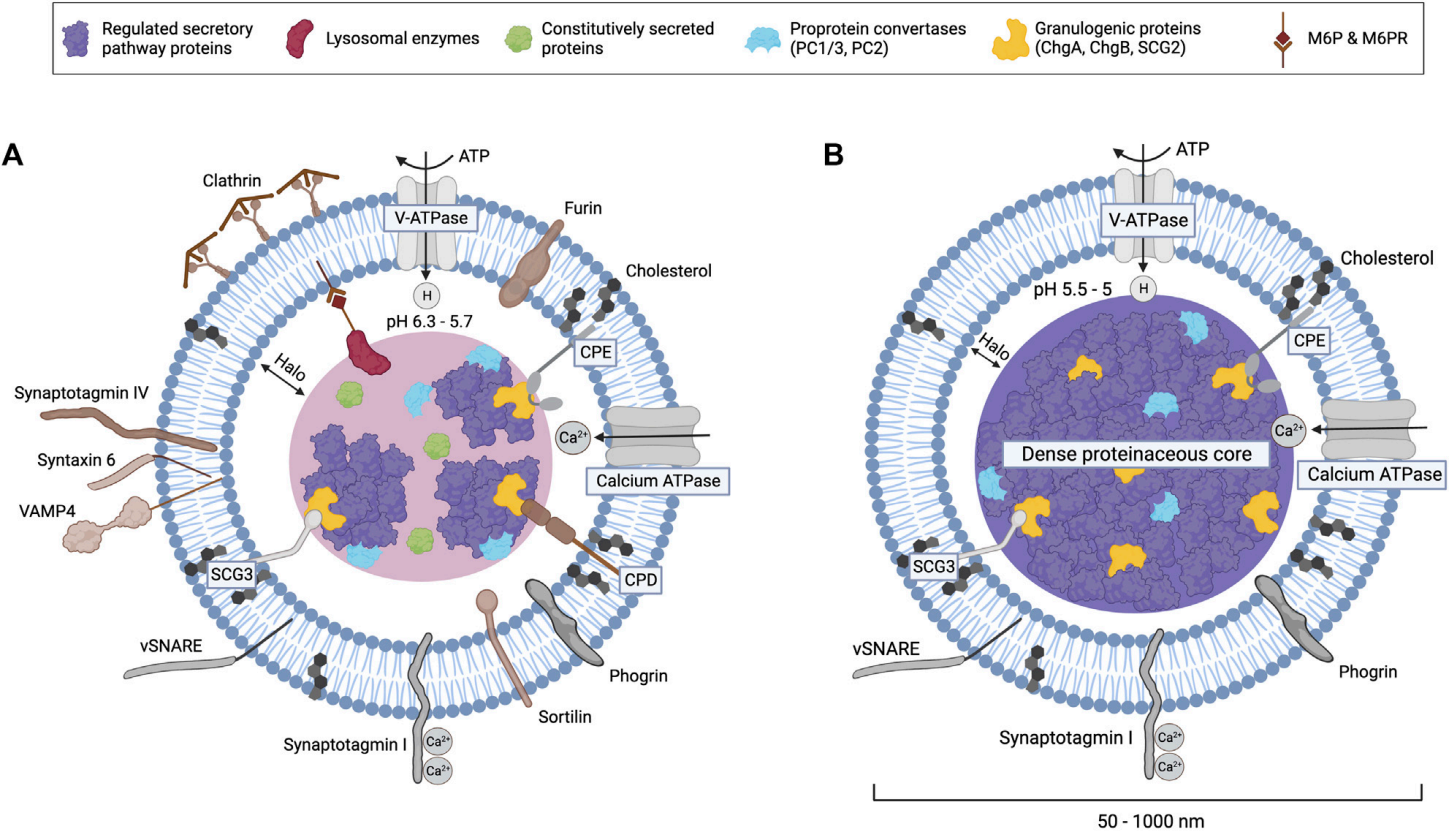
Components of immature **(A)** and mature **(B)** secretory granules. Lipid-raft-associated proteins like CPE, CPD, and secretogranin III interact with aggregates of regulated secretory pathway proteins and granulogenic proteins (e.g., granins like ChgA and ChgB) that form the dense proteinaceous core of mature SGs. Proton pumps increasingly acidify the SG lumen during maturation, which activates proprotein convertases and carboxypeptidases that process prohormones. The budding of clathrin-coated constitutive-like vesicles from immature SGs removes missorted constitutively secreted proteins and many other proteins shown in brown, including the peptidase furin, M6P-lysosomal enzymes bound to mannose-6-phosphate receptors (CI-M6PR or CD-M6PR), sortilin, synaptotagmin IV, VAMP4, and syntaxin 6. Calcium binding to synaptotagmin 1 stimulates exocytosis, which is mediated by v-SNARE proteins and other complexes. Mature SG size ranges from 50 nm in the sympathetic nervous system to 1,000 nm in pituitary mammotrophs or neurohypophyseal cells.

During granule maturation, the content and membrane composition of granules undergo remodeling (Figure 2). Homotypic fusion of ISGs, which contributes to the increased size and density of mature granules, is mediated by several proteins, including NSF, α-SNAP, syntaxin 6, and synaptotagmin IV (Tooze et al., 1991; Urbe et al., 1998; Wendler et al., 2001; Ahras et al., 2006; Kogel and Gerdes, 2010). During maturation, the lumen of ISGs progressively acidifies through the activity of vacuolar-type H^+^-ATPases (V-ATPases), which are integral membrane proteins in SGs (Figure 2) (Urbe et al., 1997; Jefferies et al., 2008). Besides enabling further condensation of soluble cargo, protein aggregation, and dense core formation, the acidic intragranular pH also activates PC1/3 and PC2 (Kogel and Gerdes, 2010). These proprotein convertases (PCs) and carboxypeptidase E (CPE) process most prohormones and neuropeptides into their mature, bioactive forms (Steiner, 1998). The maturation of ISGs also involves the removal of excess membranes and other proteins, including sortilin, carboxypeptidase D (CPD), syntaxin 6, VAMP-4, synaptotagmin IV, furin, and mannose-6-phosphate receptors (MPRs), which can be recycled back to the TGN by retrograde transport (Figures 1, 2, 4) (Klumperman et al., 1998; Varlamov et al., 1999; Eaton et al., 2000; Wendler et al., 2001; Ahras et al., 2006; Mitok et al., 2022). Mechanistically, ISGs contain coat protein patches of clathrin and AP-1 adaptor complex that mediate the budding of these proteins from ISGs into constitutive-like vesicles (Dittie et al., 1996; Dittie et al., 1997; Klumperman et al., 1998; Tooze, 1998; Eaton et al., 2000; Kakhlon et al., 2006). Overall, the process of maturation refines the composition of SGs and imparts responsiveness for regulated exocytosis (Burgoyne and Morgan, 2003; Kogel and Gerdes, 2010).

Mature SGs are stored near the membrane in the F-actin-rich cell cortex until receiving a stimulus for exocytosis. In contrast to constitutive secretion, exocytosis fusion is confined to specific sites within the plasma membrane of the polarized secreting cell and is temporally regulated by an extracellular secretion signal that increases the intracellular concentration of Ca^2+^ or cAMP (Meldolesi, 2002; Burgoyne and Morgan, 2003; Tanguy et al., 2016). Then, mature SGs undergo a series of ATP-dependent processes, such as priming, tethering, docking, and fusion to release their contents (Burgoyne and Morgan, 2003; Tanguy et al., 2016). These processes are mediated by several evolutionarily conserved proteins, including soluble N-ethylmaleimide-sensitive factor attachment protein receptors (SNAREs), NSF with adaptor proteins, Rab GTPases, SM (sec1/munc18-like) proteins, and synaptotagmins (Gerber and Sudhof, 2002; Burgoyne and Morgan, 2003; Wickner and Schekman, 2008). SNARE proteins enable the fusion of SGs with the plasma membrane through the formation of the SNARE complex, where v-SNARE (VAMP) on the SG membrane interacts with t-SNAREs (syntaxin 1 and SNAP-25) on the plasma membrane (Gerber and Sudhof, 2002; Burgoyne and Morgan, 2003). Synaptotagmins are calcium-binding proteins that act as calcium sensors and interact with both the granule and plasma membranes to trigger fusion and exocytosis (Sudhof, 2002).

Secretion is followed by rapid retrieval of the SG membrane and resident proteins through multiple endocytic pathways whose regulation is less understood but recently attracted our attention by the serendipitous discovery of the role of MAGEL2 in retromer-dependent retrograde transport (Chen et al., 2020). In the next section, we will describe the composition of SGs with a focus on the SG-unique resident proteins (i.e., granins, PCs, and CPE). Then, we will describe the current understanding of their sorting into the regulated secretion pathway at the TGN (i.e., anterograde transport) and their retrieval and sorting at the endosome (i.e., retrograde transport).

## 3 Secretory granule composition with a focus on SG-resident proteins

SGs are distinct organelles of endocrine and neuroendocrine cells with a lipid bilayer that encases a dense proteinaceous core to efficiently store hormones and neuropeptides in an osmotically inert environment (Figure 2) (Burgess and Kelly, 1987). Compared to other biological membranes, SGs have a low protein-to-lipid ratio. Membrane proteins in SGs include transporters [e.g., V-ATPase, monoamine transporter, and peptidylglycine α-amidating monooxygenase enzyme (PAM)] and proteins that facilitate SG transport and exocytosis (e.g., VAMP and synaptotagmins) (Figure 2) (Thiele and Huttner, 1998b). The composition and size of SGs vary depending on cell type and maturation state (Hammel et al., 2010; Shitara et al., 2020). SGs contain several resident proteins, including PCs, CPE, and granins, that are required for the proper sorting of cargo proteins into ISGs at the TGN and the anterograde transport of SGs.

In neurons, SGs coexist with SVs that are filled locally with neurotransmitters, such as biogenic amines, at the presynaptic terminals and regenerated after exocytosis through the refilling of their cargo. However, the vesicles themselves are generated at the TGN (Burgoyne and Morgan, 2003). Though many proteins involved in regulated secretion are shared between vesicles and granules, some proteins are specific to SVs or SGs and facilitate their distinct functions of neurotransmitter or neuropeptide/hormone secretion, respectively (Gerber and Sudhof, 2002). SG-specific components are mainly enzymes and proteins that enable neuropeptide maturation and condensation during maturation for long-term storage. Importantly, protein recycling of many of these SG-resident proteins and granule membrane components is necessary for regulated secretion in the hypothalamus (Hurtley, 1993; Bittner et al., 2013; Chen et al., 2020).

### 3.1 Proprotein convertases (PCs)

**FIGURE 3:**
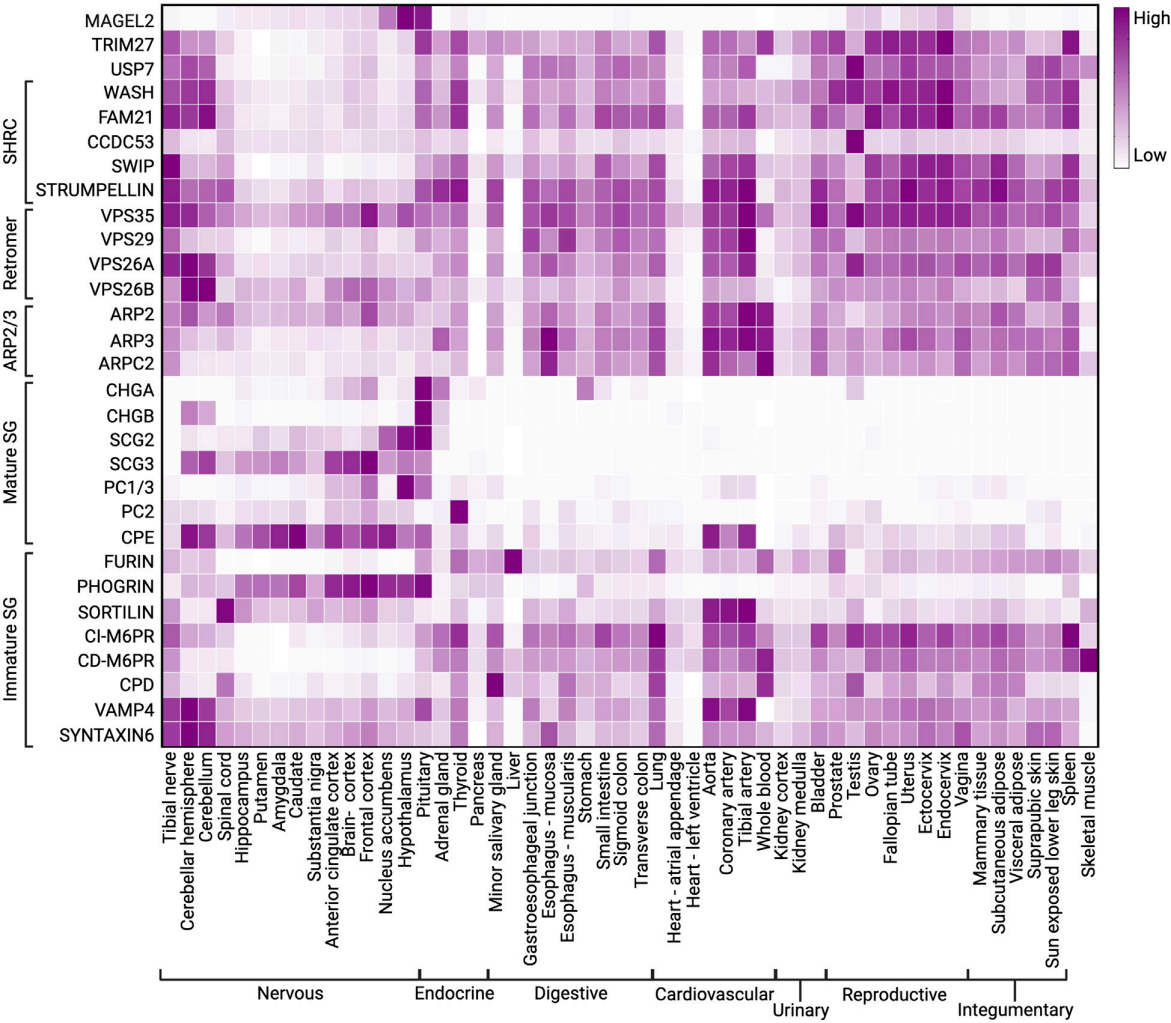
Heatmap showing expression of constitutive components of SGs, retromer, MUST, WASH, and ARP2/3 complexes. Data was extracted from GTEx on 05/24/2023.

Neuropeptide precursors packaged into SGs are cleaved into active peptides and hormones by SG-resident PCs. The PC family contains nine members: PC1/3, PC2, furin, PC4, PC5/6, PACE4, PC7, SKI-1/S1P, and PCSK9 (Seidah et al., 2013). PC1/3 and PC2 (proprotein convertase subtilisin/kexin type 1/3 and 2) are encoded by *PCSK1* and *PCSK2* genes and act as basic proprotein convertases that cleave after polybasic clusters (Seidah et al., 1991; Cendron et al., 2023). PC1/3 and PC2 are selectively expressed in endocrine and neuroendocrine cells, suggesting they are important in prohormone processing within SGs (Figure 3) (Halban and Irminger, 1994; Zhou et al., 1999). PC1/3 and PC2 process a plethora of prohormones, including pro-opiomelanocortin (POMC), neuropeptide Y (NPY), agouti-related peptide (AGRP), progrowth-hormone releasing hormone (GHRH), prothyrotropin-releasing hormone (TRH), proinsulin, and proglucagon (Paquet et al., 1996; Nillni, 2010; Seidah, 2011; Cawley et al., 2016). Consequently, defects in PC1/3 or PC2 activity result in several endocrinopathies in both humans and rodents (Furuta et al., 1997; Jackson et al., 1997; Steiner, 1998; Zhu et al., 2002; Scamuffa et al., 2006; Mbikay et al., 2007; Anini et al., 2010; Creemers et al., 2012; Seidah and Prat, 2012).

### 3.2 Carboxypeptidase E (CPE)

After endoproteolytic cleavage by PC1/3 and/or PC2, the newly exposed C-terminal basic residues of prohormones are removed by CPE, another resident protein of SGs (Burgoyne and Morgan, 2003). CPE was first identified as enkephalin convertase and subsequently found to cleave the C-terminally extended basic residues from diverse peptide intermediates, including POMC and probrain-derived neurotrophic factor (BDNF) (Fricker and Snyder, 1982; Hook et al., 1982; Fricker, 1988; Lou et al., 2005). CPE is expressed primarily in endocrine tissues and specific areas of the central nervous system (Figure 3) (Fricker, 1988; Cawley et al., 2012; Ji et al., 2017). CPE differs from other carboxypeptidases in that its optimal pH is in the acidic range, consistent with its localization to acidic compartments of the TGN and to the dense core of SGs where prohormone processing occurs (Supattapone et al., 1984; Ji et al., 2017).

Like other proteins in the regulated secretory pathway, CPE is synthesized in the ER as a 476-amino acid precursor containing an N-terminal signal peptide that directs proCPE into the ER before its removal (Song and Fricker, 1995a). ProCPE is transported through the Golgi to SGs where the 17-amino acid “pro” region is cleaved after a penta-arginine sequence to generate mature membrane-bound CPE that is glycosylated at two N-linked glycosylation consensus sites, Asn139 and Asn390 (Song and Fricker, 1995a; Cawley et al., 2012; Ji et al., 2017). Within SGs, the membrane-bound CPE can be further processed by cleavage of its C-terminal cytoplasmic tail to generate a soluble form of CPE that is enzymatically more active (Hook, 1985; Fricker and Devi, 1993).

In addition to its enzymatic function, CPE acts as the quintessential regulated secretory pathway sorting receptor for many prohormones (e.g., POMC, pro-BDNF, proenkephalin, proinsulin, and prophogrin). Under mildly acidic conditions and increasing calcium concentrations, CPE aggregates and binds SG cargo proteins through their prohormone sorting signals, such as those found in POMC (Song and Fricker, 1995b; Rindler, 1998; Zhang et al., 1999). CPE binding enables protein condensation and directs cargo proteins to SGs (Cawley et al., 2012). The C-terminus of CPE forms an amphipathic α-helix under acidic conditions that binds to lipid rafts in the TGN membrane and directs prohormones bound to CPE into nascent granules (Fricker et al., 1990; Dhanvantari and Loh, 2000; Dhanvantari et al., 2002; Zhang et al., 2003; Cawley et al., 2012). In a neutral environment (pH 7.2), CPE’s C-terminus does not exhibit a helical secondary structure, preventing premature association with the membrane (Dhanvantari et al., 2002). Interestingly, CPE may only sort certain prohormones to the regulated secretory pathway, as CPE depletion did not affect sorting of chromogranin A (CHGA) (Cool et al., 1997; Normant and Loh, 1998; Ji et al., 2017).

### 3.3 The granin family

Chromogranins A and B (CHGB), secretogranins II and III (SCGII and SCGIII), and a few additional related proteins together comprise the granin family of water-soluble acidic glycoproteins (Bartolomucci et al., 2011). These granin proteins serve essential roles in the regulated secretory pathway, with the chromogranins comprising much of the SG matrix (O'Connor and Frigon, 1984; Borges et al., 2010), and, accordingly, are predominantly expressed in endocrine and neuroendocrine cells (Figure 3) (Day and Gorr, 2003; Dominguez et al., 2018). Like CPE and other SG resident proteins, granins are also synthesized at the rough ER, inserted into the ER cisternae via a signal peptide located at their N termini, and trafficked to the TGN via transport vesicles (Bartolomucci et al., 2011). Several biochemical properties that are critical for the function of granins include an acidic isoelectric point, Ca^2+^ binding, and thermostability (Yoo and Albanesi, 1991; Taupenot et al., 2003; Bartolomucci et al., 2011). Additionally, granins aggregate in an acidic environment (pH 5.5) with a millimolar concentration of calcium ions and, by doing so, induce granule formation (Chanat and Huttner, 1991; Parmer et al., 1993; Koshimizu et al., 2010). Granins are negatively charged, which may prevent premature aggregation, but a surplus of calcium ions and protons in the SG lumen may help neutralize the repulsive forces among the granin proteins to allow aggregation (Glombik and Gerdes, 2000). An alternative explanation proposed is that the pH gradient prompts interactions between negative and positive charges of cargo proteins, and then divalent ions may generate a chelate bridge between two negatively charged granins to permit aggregation (Ma et al., 2008; Zhang et al., 2010).

CHGA, the most well-studied granin family member, is a prohormone and a granulogenic factor in neuroendocrine tissues (Laguerre et al., 2020). While CHGA is mostly hydrophilic, its C- and N-termini contain hydrophobic and cell-specific evolutionarily conserved sequences necessary for sorting and granulogenesis (Yoo and Lewis, 1993; Cowley et al., 2000; Montero-Hadjadje et al., 2009; Elias et al., 2010). Cysteine residues within the amino terminus form an intramolecular disulfide loop to interact with the membrane (Parmer et al., 1993; Yoo, 1994; Kang and Yoo, 1997). The primary structure of CHGA contains several glutamic acid stretches that can interact with Ca^2+^, leading to aggregation in specific environments, such as the TGN and SG (Parmer et al., 1993). CHGA may associate with membrane either directly by binding to specific lipids (e.g., phosphatidic acids enriched in TGN and SG membranes) (Carmon et al., 2020; Tanguy et al., 2020) or indirectly by interacting with SCGIII, which in turn binds to cholesterol-rich membranes and targets proteins to the regulated secretory pathway (Hosaka et al., 2004; Han et al., 2008).

Besides their granulogenic function, granins contribute to calcium homeostasis and many are also precursors of bioactive peptides that, upon further processing in ISGs, modulate different physiological processes, including pain pathways, inflammatory responses, metabolic and mood disorders, and blood pressure (Montero-Hadjadje et al., 2008). As an example, the CHGA-derived peptide pancreastatin, which was the first granin-derived peptide discovered, strongly inhibits glucose-induced insulin release (Tatemoto et al., 1986; Bartolomucci et al., 2011).

Even though the granin family is ubiquitously expressed in neuroendocrine tissues, individual proteins exhibit tissue specificity and redundancy (Figure 3). For example, the ablation of *Chga* and *Chgb* reduced the size and number of SGs in adrenal chromaffin cells, while SGs in mouse hippocampal neurons were unaffected (Dominguez et al., 2018). In addition, ablation of *Chga* upregulated the expression of Chgb and secretogranins II-VI in the adrenal medulla and other endocrine glands (Hendy et al., 2006). These data indicate that chromogranin-mediated dense core formation of SG is tissue-specific and that other granin family members may perform a similar function.

### 3.4 Mannose-6-phosphate receptors (MPRs)

MPRs facilitate the transport of soluble acid hydrolases from the TGN to the lysosome by binding to mannose-6-phosphate (M6P) modifications on the enzymes. There are two different MPRs, the larger cation-independent receptor (CI-MPR) and the smaller cation-dependent receptor (CD-MPR) that binds M6P more efficiently in the presence of divalent cations (Gary-Bobo et al., 2007). Besides binding to phosphomannosyl residues, the extracellular region of CI-MPR, also known as IGF2R, binds insulin-like growth factor (IGF)-II to facilitate its endocytosis and clearance by lysosomal degradation (Oka et al., 1985; Oshima et al., 1988; Schmidt et al., 1995; Brown et al., 2008). CI-M6PR also binds other ligands, such as retinoic acid, granzyme B, latent TGF-β, urokinase-type plasminogen activator receptor, and leukemia inhibitory factor, impacting a variety of biological pathways (Purchio et al., 1988; Kang et al., 1997; Blanchard et al., 1999; Godar et al., 1999; Veugelers et al., 2006).

**FIGURE 4:**
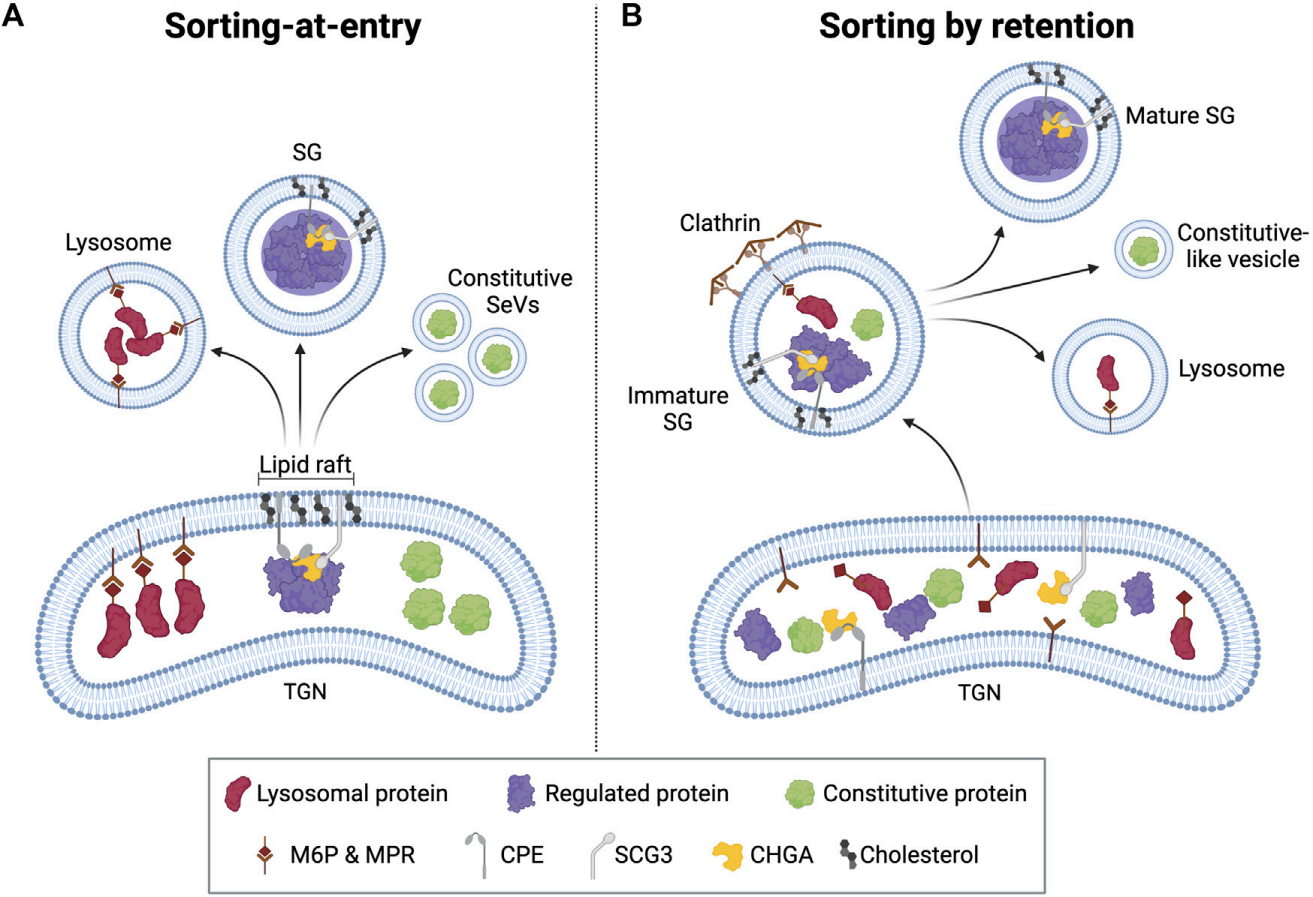
Proposed sorting models for secreted proteins. **(A)** In the “sorting for entry” model, secreted and lysosomal proteins are segregated by binding to specific receptors clustered in the TGN before granule formation. **(B)** In the “sorting by retention” model, secreted and lysosomal proteins enter nascent SGs, but the non-regulated secretory proteins are excluded from the maturing SG by budding, possibly mediated by clathrin.

**FIGURE 5:**
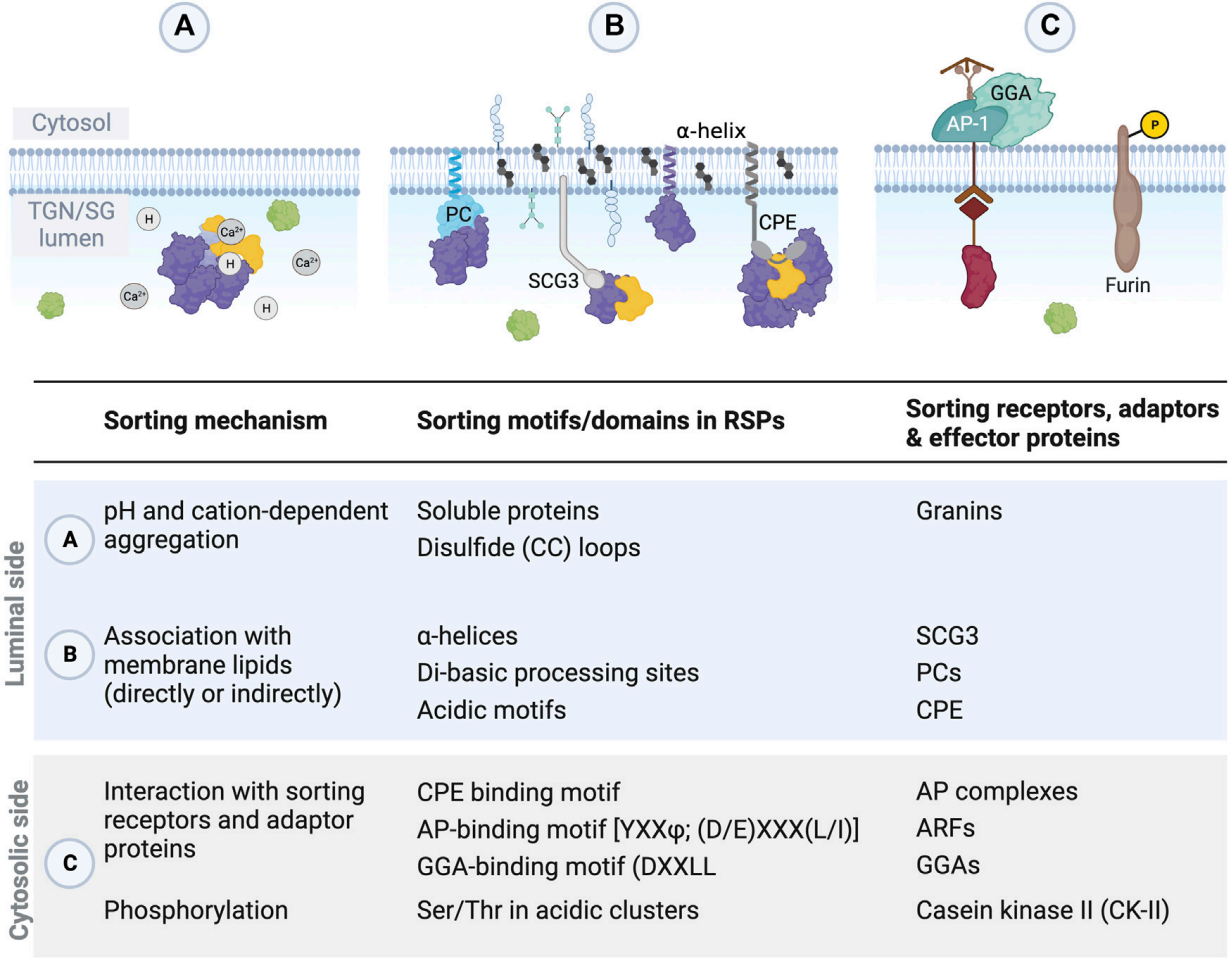
Sorting of proteins destined for the regulated secretory pathway occurs through various mechanisms, motifs, and adaptor proteins. **(A,B)** Within the lumen of the TGN and ISGs, sorting motifs within RSPs and interactions with other proteins facilitate aggregation and association with the membrane. **(C)** On the cytosolic side of the TGN/SG membrane, adaptor proteins recognize specific motifs in RSPs to help with sorting, and phosphorylation of some RSPs, like furin, enhances sorting.

CI-MPR is one of the most thoroughly studied proteins that is removed from ISGs and then recycled back to the TGN (Figures 1, 2, 4, 5) (Bonnemaison et al., 2013; Seaman, 2018). As SGs mature, the concentration of MPRs declines by about 90% (Klumperman et al., 1998). MPRs are sorted from ISGs by binding to adaptor protein 1 (AP-1) and the Golgi-localized, γ-ear containing, ARF-binding (GGA) family of proteins in clathrin and syntaxin 6–positive vesicles that are delivered to endosomes (Figures 1, 2) (Klumperman et al., 1998; Coutinho et al., 2012). Then, the multiprotein retromer complex mediates the endosome-to-TGN retrieval of CI-MPR (Bonnemaison et al., 2013; Seaman, 2018). Retromer is critical for the recycling and reuse of MPRs in a new cycle of acid hydrolase transportation.

In the following sections, we discuss sorting into the regulated secretory pathway, as well as the retromer-dependent recycling of CI-MPR, potential retrograde trafficking targeting motifs, and finally, the tissue-specific role of MAGEL2 in the retromer-dependent recycling of mature and immature SG components.

## 4 Sorting compartments, mechanisms, and signals in the anterograde pathway of regulated secretion

Overall, the cellular life of regulated secretory proteins (RSPs) begins similarly to constitutive secretory proteins. An N-terminal signal sequence enables the signal recognition particle (SRP)-dependent co-translational translocation into the ER lumen. This signal sequence is then removed from the nascent protein, and the protein is post-translationally modified during anterograde transport through the Golgi to the TGN. The TGN serves as the main sorting station in the anterograde traffic of secretory proteins, and the sorting is fine-tuned during ISG maturation when the missorted proteins are removed by clathrin-coated vesicles (Figures 1, 2, 4) (Farquhar and Palade, 1998). The canonical targeting signals that direct proteins into the constitutive secretory pathway or to other cellular destinations (e.g., the plasma membrane, mitochondria, nucleus, and lysosomes), as well as the signals for ER or Golgi retention, are well established. In contrast, RSPs are not targeted to SGs by a common sorting motif but rather through intrinsic protein features and a variety of motifs that may synergize to increase granule-sorting efficiency (Glombik and Gerdes, 2000; Lacombe et al., 2005). As an example of multiple sorting signals being present on a single molecule, prothyrotropin-releasing hormone has two intermediates that are stored in different vesicles and secreted by different stimuli (Perello et al., 2008). Specific sorting signals that have been discovered on RSPs include linear amino acid sequences, conformation epitopes, polypeptides, and post-translational modifications (Thiele and Huttner, 1998b). The propensity of RSPs to aggregate contributes to their sequestration in the TGN, packaging into ISGs, and removal of mistargeted proteins from ISGs during maturation. Besides aggregation, association with distinct membrane lipids, sorting receptors, and adaptor proteins also facilitate the sorting of soluble RSPs to SGs (Thiele and Huttner, 1998b; Glombik and Gerdes, 2000; Kogel and Gerdes, 2010; Bonnemaison et al., 2013). These sorting signals and mechanisms can also be tissue- or cell-specific (Chidgey, 1993; Marx et al., 1999; Cowley et al., 2000; Dikeakos and Reudelhuber, 2007).

### 4.1 Sorting models

For secreted proteins, there are two proposed sorting models that are not mutually exclusive, as supporting evidence suggests both models may occur in cells (Figure 4) (Dikeakos and Reudelhuber, 2007; Goronzy and Weyand, 2009). In the “sorting-at-entry” model, constitutively secreted proteins are segregated from regulated proteins by binding to specific receptors clustered in the TGN before granule formation (Figures 4, 5) (Chidgey, 1993). Initial aggregation of cargo proteins (e.g., granins and prohormones) in a mildly acidic pH (6.0–6.5) and cation-dependent manner excludes certain constitutive proteins. Then, the aggregates can bind to sorting receptors at the TGN membrane. One of the first sorting receptors proposed was CPE, which sorts POMC and proBDNF (Cool et al., 1997; Lou et al., 2005). SCGIII can also act as a sorting receptor, as SCGIII associates with cholesterol-sphingolipid-rich membrane microdomains (i.e., lipid rafts) in the TGN membrane and, by doing so, serves as a sorting receptor for CHGA in pituitary and pancreatic cells (Tooze, 1998; Hosaka et al., 2004; Park and Loh, 2008). Receptors are then recycled through vesicles budding off ISGs (Tooze, 1998).

In the “sorting by retention” model, both regulated and non-regulated proteins enter the nascent granule with the latter proteins excluded from the ISG by budding of clathrin-coated vesicles (Chidgey, 1993). In support of this model, lysosomal proteins have been found in immature SGs, which are then most likely excluded through binding to MPRs and budding of a constitutive-like vesicle. All evidence points to the conclusion that the best-fitting sorting model is reliant on the specific protein, its affinity for aggregation, the relative speed of its synthesis, and tissue or cell specificity (Tooze, 1998). As an example of cell specificity, the sorting domain in the C-terminus of PC2 is essential for sorting in Neuro2A cells (Assadi et al., 2004) but is not required in corticotrophic AtT-20 cells (Taylor et al., 1998; Lacombe et al., 2005). Similarly, the SG sorting of CHGA and CHGB requires an N-terminal domain in neuroendocrine PC12 cells but not in endocrine GH4C1 cells (Chanat et al., 1993); rather, a C-terminal region of CHGA was required for proper sorting in GH4C1 cells (Cowley et al., 2000). Different sorting domains on the same protein offer insights not only into targeting efficiency but also into the possibility of targeting signal redundancy, which may offer protection from mutations and other damage.

### 4.2 Sorting mechanisms

Mechanistically, several processes contribute to sorting RSPs into the regulated secretory pathway. An intrinsic propensity to aggregate combined with Ca^2+^-binding motifs underly one sorting mechanism–selective aggregation of a subset of soluble proteins in the TGN, excluding soluble non-aggregating proteins and giving rise to SGs (Figure 5). In this aggregation mechanism, only one or perhaps a small number of proteins need to interact with the membrane, as the other regulated proteins are targeted to the SG through protein-protein interactions mediated by helper proteins, like the granins. Accordingly, overexpression of CHGB led to more effective sorting into granules in cells (Natori and Huttner, 1996; Huh et al., 2003). Expression of granulogenic proteins (e.g., CHGA) in regulated secretory-deficient cells was sufficient to induce the formation of vesicles that resembled SGs (Kim et al., 2001; Huh et al., 2003; Beuret et al., 2004). However, aggregation alone is insufficient to target all RSPs to SGs (Quinn et al., 1991; Jutras et al., 2000), suggesting additional sorting mechanisms (Lacombe et al., 2005).

Association with lipid rafts is another sorting-at-entry mechanism used by several RSPs (Figure 5). Through insertion into lipid rafts, the transmembrane domains of several prohormone-processing enzymes, like PAM, mediate their own sorting into the regulated secretory pathway (Bell-Parikh et al., 2001). In addition, several sorting receptors in the TGN recruit and fasten SG-destined proteins to membrane sites where a nascent vesicle will bud (Park and Loh, 2008). For example, SCGIII, CPE, PC1/3, and PC2 have all been reported to associate with cholesterol- and sphingolipid-rich lipid rafts, which is crucial for their own targeting to the regulated secretory pathway (Dhanvantari and Loh, 2000; Jutras et al., 2000; Arnaoutova et al., 2003; Assadi et al., 2004; Hosaka et al., 2004; Dikeakos et al., 2009).

Besides interacting with sorting receptors, various cargo adaptors, such as adaptor protein (AP) complexes and monomeric GGA proteins, can help direct proteins into their appropriate transport carriers (Figure 5) (Robinson, 2004). The AP family includes five cytosolic heterotetrameric complexes, AP-1 to AP-5, that mediate sorting of transmembrane proteins on defined intracellular routes (Bonnemaison et al., 2013). AP-1, AP-3, and AP-4 are associated with the TGN, and AP-1 also removes material from ISGs (Bonnemaison et al., 2013; Guardia et al., 2018). AP-2 regulates clathrin-mediated endocytosis at the plasma membrane, and AP-5 facilitates the retrograde transport of proteins from endosomes to the TGN (Bonnemaison et al., 2013; Hirst et al., 2013; Guardia et al., 2018). The subunits of these AP complexes have several isoforms in mammals, suggesting evolutionary adaptation to finetune the process of regulated secretion, particularly in neurons that uniquely express two AP-3 subunits (Dell'Angelica et al., 1997; Boehm and Bonifacino, 2002; Bonifacino, 2014; Li et al., 2016; Guardia et al., 2018). GGA proteins act as monomeric clathrin adaptors (Bonifacino, 2004). Arf small G proteins in an active GTP-bound, membrane-associated state mediate the membrane recruitment of AP complexes and GGAs (Traub et al., 1993; Austin et al., 2000; Collins et al., 2003; Ren et al., 2013). Then, AP complexes can bind to the cytoplasmic tails of cargo proteins and recruit coat proteins (i.e., clathrin) and accessory proteins to drive vesicle formation (Bonnemaison et al., 2013; Tan and Gleeson, 2019). Upon the release of secretory vesicles from the TGN membrane, coat proteins are dissociated and recycled for additional rounds of vesicle formation (Tan and Gleeson, 2019).

### 4.3 Sorting domains and motifs

The motifs and domains on RSPs that are responsible for their proper sorting into SGs remain enigmatic and not uniform. In general, sorting signals facilitate the aggregation of cargo proteins or piggyback anchoring through association with membrane domains and other proteins (Figure 5).

Within the TGN lumen, proteins targeted to SGs tend to aggregate, not only promoting formation of the dense core of the SGs but also enabling their own sorting to the regulated secretion pathway (Figure 5) (Burgess and Kelly, 1987). Several motifs and domains within RSPs promote aggregation and sorting to SGs. In the case of CHGA, several glutamic acid repeats interact with Ca^2+^ and promote aggregation at the TGN and in SGs (Parmer et al., 1993). Small disulfide (CC) loops also act as SG sorting signals by promoting self-aggregation at the TGN (Reck et al., 2022). These CC loops are present frequently at the very N- or C-terminus of proteins or close to processing sites and thus potentially exposed (Reck et al., 2022). For example, in POMC, an N-terminal 13-residue CC loop is necessary and sufficient for granule sorting (Tam et al., 1993; Cool et al., 1995; Loh et al., 2002). Longer disulfide loops in CHGA and CHGB are also involved in their sorting to SGs (Kang and Yoo, 1997; Kromer et al., 1998; Glombik et al., 1999; Taupenot et al., 2002). In addition, di-basic processing sites and acidic motifs in prohormones were shown to promote granule sorting, likely via interaction with PCs or CPE (Brechler et al., 1996; Lou et al., 2005). The sorting signal motif for CPE recognition was first identified as two acidic residues and two aliphatic hydrophobic residues in POMC (Cool et al., 1995), and similar sorting motifs were subsequently found in proinsulin (Dhanvantari et al., 2003), BDNF (Lou et al., 2005), and proenkephalin (Normant and Loh, 1998; Cawley et al., 2016).

Amphipathic helices enable interaction with the granule membrane and are important for the incorporation of RSPs and enzymes, like PCs and CPE, into SGs. Besides a disulfide-bonded hydrophobic loop that interacts with SG membrane (Kang and Yoo, 1997), the N-terminal region of CHGA also contains an amphipathic α-helix that may be necessary for sorting CHGA/hormone aggregates into SGs (Thiele and Huttner, 1998a; Taupenot et al., 2002; Laguerre et al., 2020). Similarly, proglucagon is sorted by α-helices present in the mature hormone domains rather than in the typical prohormone domain (McGirr et al., 2013; Guizzetti et al., 2014). Tissue-specific processing of proglucagon by PCs yields glucagon in pancreatic α cells (Furuta et al., 2001) or glucagon-like peptide 1 (GLP-1) and GLP-2 in intestinal L cells and hypothalamic neurons (Dhanvantari et al., 1996; Damholt et al., 1999; Holt et al., 2019). Although each of these products contains α-helices, only the non-amphipathic, dipolar α-helices on glucagon and GLP-1 efficiently target them to the regulated secretory pathway (Guizzetti et al., 2014). In contrast, GLP-2 has a more uniform negative charge distribution along the length of its α-helix (Guizzetti et al., 2014). PC1/3, PC2, and PC5/6A also contain α-helices in their C-termini that form electrostatic interactions and help sort proteins into the regulated secretion pathway (Assadi et al., 2004; Dikeakos et al., 2007b; Dikeakos et al., 2009). There seems to be no correlation between the helix length or the isoelectric point and sorting efficiency, but helices with a positive or negative charge and a hydrophobic segment seem the most effective in sorting (Dikeakos et al., 2007a), suggesting electrostatic interactions also play a role in aggregation, either inter- or intra-molecularly (Zhang et al., 2010). Indeed, Ma et al. (2008) identified four to five residues, two of which are charged, as the elementary sorting unit for protachykinin targeting to SGs. Clustering of these charged elementary units improved aggregation, leading to an additive and graduated effect that also improved sorting (Ma et al., 2008). In summary, charged amphipathic helices or non-amphipathic helices that have segregated charges and a hydrophobic patch are sufficient for targeting to secretory granules (Dikeakos et al., 2007a).

On the cytosolic side, specific motifs in the cytoplasmic domains of SG membrane proteins enable binding to adaptor proteins (Figure 5). The acidic-cluster-dileucine motif (DXXLL, where X is any amino acid) found in MPRs and sortilin, for example, is recognized by GGA proteins (Johnson and Kornfeld, 1992a; b; Chen et al., 1997; Nielsen et al., 2001; Puertollano et al., 2001). Serine and threonine residues embedded within cytosolic acidic clusters serve as substrates for casein kinase II (CKII) phosphorylation that enhances the sorting of proteins, like PAM, CI-MPR, and furin (Meresse et al., 1990; Jones et al., 1995; Chen et al., 1997; Steveson et al., 2001). In addition, VAMP4, phogrin, MPRs, and lysosomal proteins contain tyrosine-based (YXXΦ, where Φ is a bulky hydrophobic residue) and dileucine [(D/E)XXXL(L/I)] sorting motifs that are recognized by AP complexes (Glickman et al., 1989; Honing et al., 1997; Peden et al., 2001; Ghosh and Kornfeld, 2004; Torii et al., 2005; Wasmeier et al., 2005; Braulke and Bonifacino, 2009). These interactions between sorting motifs and adaptor proteins directs MPRs and their cargo proteins into TGN-derived, clathrin-coated vesicles that fuse with endosomes (Doray et al., 2002; Waguri et al., 2003; Braulke and Bonifacino, 2009).

In summary, several years of research uncovered how cells control the secretion of proteins and proposed two major models, sorting-at-entry and sorting by retention, to describe how proteins are sorted into the proper pathway at the TGN. Both models incorporate several sorting mechanisms that promote protein aggregation and binding to unique membrane lipids, leading to SG formation. After budding from the TGN, both constitutive and regulated secretory vesicles are transported to secretion sites at the plasma membrane via microtubule-based transport systems (Park and Loh, 2008).

### 4.4 Cytoskeletal filaments in the sorting and trafficking of regulated proteins

Kinesin, dynein, and myosin are molecular motors that transport SeVs and SGs along microtubule or actin tracks. In general, SeVs and SGs use the same type of microtubule motor, such as kinesin, for anterograde transport to the secretion sites, and cytoplasmic dynein for retrograde transport back to the cell body (van den Berg and Hoogenraad, 2012). Tight regulation of the transport machinery is critically important to ensure that proteins are picked up and delivered to the right place at the right time. At the end of microtubule-based transport, SGs are transferred to the actin cortex close to the plasma membrane with the help of myosin V and the F-actin motor proteins (Rose et al., 2003; Rudolf et al., 2003). Actin and myosin have established themselves as key players in regulated secretion by providing tracks to target SGs to fusion sites, actively squeezing cargoes from fused vesicles, and following fusion, retrieving excess membrane to maintain cell surface area and recycle several SG-resident proteins (Rudolf et al., 2003; Rojo Pulido et al., 2011; Li et al., 2018; Miklavc and Frick, 2020). Besides its role as the transport platform for myosin motors, F-actin acts as a physical barrier for SG exocytosis and is also involved in the regulation of sorting at the TGN (Park and Loh, 2008; Gutierrez and Villanueva, 2018). During SG biogenesis, F-actin is recruited to the budding granule by actin-related protein-2/3 (ARP2/3) and myosin 1b (Delestre-Delacour et al., 2017). ARP2/3 complex binds to actin and exerts an active role in SG formation through its nucleation and branching activities that provide a structural or force-generating scaffold (Goley and Welch, 2006). The nucleation core activity of ARP2/3 is activated by nucleation promoting factors, such as members of the Wiskott-Aldrich syndrome family (WASP, N-WASP, WAVE, and WASH proteins) (Welch and Mullins, 2002; Alekhina et al., 2017).

**FIGURE 6:**
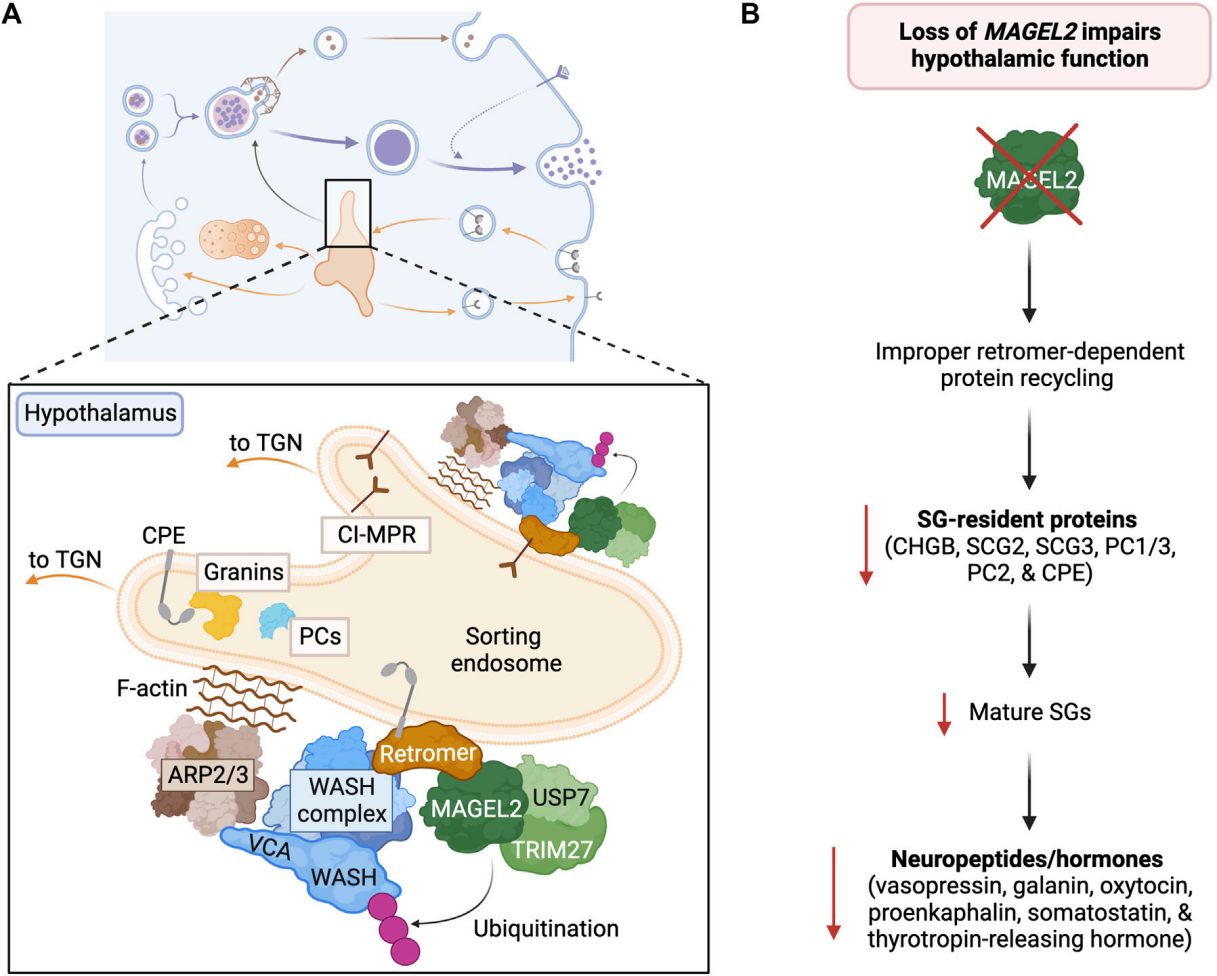
MAGEL2 functions in regulated secretion of the hypothalamus. **(A)** Within hypothalamic neurosecretory cells, MAGEL2 plays a critical role in the retromer-mediated transport of SG components (i.e., PC1/3, PC2, CHGA, CHGB, and CPE) and the lysosomal CI-MPR from the sorting endosome to the TGN. MAGEL2-TRIM27-mediated ubiquitination leads to WASH activation and actin nucleation. **(B)** The loss of *MAGEL2* leads to decreased abundance of SGs, SG-resident proteins, and neuropeptides in the hypothalamus, thus impairing hypothalamic neuroendocrine function.

The fine-tuned control of actin polymerization on endosomes is fundamental for the retrieval and recycling of several cargoes (Puthenveedu et al., 2010; Simonetti and Cullen, 2019). Retrieval and recycling are orchestrated by several multi-protein complexes, including retromer, commander/CCC/retriever, sorting nexins, and the ARP2/3-activating WASH complex (Simonetti and Cullen, 2019; MacDonald et al., 2020; Placidi and Campa, 2021). Recently, MAGEL2 regulation of the WASH complex and ARP2/3 activation was shown to prevent lysosomal degradation of SG-resident proteins and enable the proper neuroendocrine function of the hypothalamus, suggesting a tissue-specific regulation of compensatory endocytosis in regulated secretion (Figure 6) (Chen et al., 2020). In the next paragraphs, we will briefly summarize retromer-dependent retrograde recycling and its regulation.

## 5 Endocytic recycling of proteins in the regulated secretory pathway

In the secretory pathway, the anterograde trafficking of membranes is compensated by the retrograde transport of lipids and proteins to maintain membrane homeostasis and recycle various proteins and lipids. Endocytic recycling contributes to membrane receptor abundance, cell resensitization, and downstream signaling (Sannerud et al., 2003; Lucas and Hierro, 2017). Protein recycling is also important in the regulated secretion of hormones and neuropeptides (Ferraro et al., 2005; Chen et al., 2020; Neuman et al., 2021).

Endocytosis, occurring through clathrin-dependent and -independent mechanisms, internalizes lipids and proteins from the plasma membrane into early/sorting endosomes (Cullen and Steinberg, 2018; MacDonald et al., 2020). From the sorting endosome, cargo can be recycled back to the plasma membrane or retrograde trafficked to the TGN; both pathways prevent the lysosomal degradation of these recycled proteins (Chamberland and Ritter, 2017; Ma and Burd, 2020; Placidi and Campa, 2021). Ubiquitinated endosomal cargo is directed to lysosomes for degradation by ESCRT (endosomal sorting complex required for transport) complexes (Placidi and Campa, 2021).

Cargo recycling was historically thought of as a passive process, but the recent identification and characterization of specialized recycling complexes (i.e., retromer and commander/CCC/retriever complexes) that recognize specific signals in cytoplasmic domains of cargo proteins revealed the complexity of retrograde sorting machinery (Cullen and Steinberg, 2018; Singla et al., 2019; MacDonald et al., 2020; Placidi and Campa, 2021; Yong et al., 2022). The discovery that several transmembrane proteins, like CI-MPR and β2-adrenergic receptor, travel specific recycling routes first suggested the existence of active sorting to direct cargo into a non-degradative endosomal pathway to its correct cellular destination, such as the plasma membrane or TGN (Duncan and Kornfeld, 1988; Cao et al., 1999; MacDonald et al., 2020; Placidi and Campa, 2021). The subsequent discovery of the trimeric retromer complex (VPS26, VPS29, VPS35) (Arighi et al., 2004; Seaman, 2004), other retromer-like complexes (i.e., retriever) (McNally et al., 2017), and the WASH complex (composed of WASH, FAM21, CCDC53, SWIP/KIAA1033, and Strumpellin) (Derivery et al., 2009; Gomez and Billadeau, 2009; Harbour et al., 2010) confirmed that sequence-dependent recycling actively opposes degradation (Gershlick and Lucas, 2017; MacDonald et al., 2020; Placidi and Campa, 2021). The WASH complex is the major endosomal actin polymerization-promoting complex that stimulates the activity of the ubiquitously expressed ARP2/3 F-actin nucleation complex and the formation of branched actin patches (Derivery et al., 2009; Gomez and Billadeau, 2009; Liu et al., 2009; Seaman et al., 2013). As in anterograde transport, actin and regulation of its polymerization are key components of retrograde transport (Puthenveedu et al., 2010; Seaman et al., 2013; Simonetti and Cullen, 2019; Miklavc and Frick, 2020). Furthermore, the mammalian-specific MAGEL2 has emerged as a tissue-specific regulator of WASH activation and actin nucleation in the hypothalamus (Figure 6) (Chen et al., 2020).

More than 150 membrane proteins rely on the retromer complex for their localization on the cell surface (Steinberg et al., 2013), and neurons are particularly susceptible to any changes in endosomal trafficking due to their long axons and branched dendrites (Chamberland and Ritter, 2017). Retromer dysfunction has been linked to neurodegenerative diseases, such as Parkinson’s disease, Alzheimer’s disease, and frontotemporal lobar degeneration (Harrison et al., 2014; Seaman and Freeman, 2014; Reitz, 2018; Zhang et al., 2018). The loss of *MAGEL2* in neurons leads to aberrant endosomal protein trafficking and reduces the abundance of SG proteins, contributing to the etiology of Prader-Willi syndrome (PWS) (Chen et al., 2020). In addition to neuronal and neuroendocrine functions, several other physiological processes depend on retromer (Burd and Cullen, 2014). Furthermore, pathogens like *Chlamydia trachomatis* and *Legionella pneumophila* hijack the retromer complex for successful infection (Elwell et al., 2017; Elwell and Engel, 2018).

To summarize, the retrograde recycling of proteins is important for maintaining a plethora of physiological functions. The proper sorting of cargo proteins is critical for returning proteins to their proper place for reuse and preventing their lysosomal degradation. Retromer is at the very center of this retrograde sorting that happens at the early endosome, soon after the cargo is endocytosed.

### 5.1 Retromer evolution and function

Retromer is an evolutionarily conserved complex that regulates the retrograde pathway across all eukaryotes (Koumandou et al., 2011; McGough and Cullen, 2011; Chamberland and Ritter, 2017). In *Saccharomyces cerevisiae*, where retromer was first discovered, five vacuolar protein sorting (VPS) proteins compose the two retromer subcomplexes: the cargo-selective complex and the tubulation complex, both of which are conserved in higher eukaryotes (Seaman et al., 1998; Neuman et al., 2021). The cargo-selective complex is a trimer of Vps26, Vps29, and Vps35, which recruits cargo via an association between Vps35 and a sorting motif located within the cytoplasmic tail of cargo (Seaman et al., 1998). Retromer is recruited to the endosomal membrane by the sorting nexin (SNX) proteins Vps5 and Vps17 that form the tubulation complex and contain C-terminal Bin/amphysin/Rvs (BAR) domains that promote membrane tubulation and cargo vesicle formation (Horazdovsky et al., 1997; Nothwehr and Hindes, 1997; Seaman et al., 1998; Chamberland and Ritter, 2017).

Compared to the yeast retromer, the mammalian retromer is more complex and allows for more cargo specificity and transport regulation (Chen et al., 2019). In addition, the interactions between the retromer subcomplexes are more transitional and may not always occur in mammalian cells (Harbour and Seaman, 2011; Cullen and Steinberg, 2018). For example, both subcomplexes are needed to facilitate recycling of CI-MPR and other cargo in yeast cells (Yong et al., 2022), whereas the SNX-BAR dimer and not the VPS trimer is required for CI-MPR recycling in mammalian cells (Kvainickas et al., 2017; Simonetti et al., 2017), implying functional segregation of the two subcomplexes (Chamberland and Ritter, 2017). The acquisition of new binding partners in mammals that are not found in yeast provides further evidence of the functional divergence between the subcomplexes (Chamberland and Ritter, 2017). From now on in this manuscript, retromer will refer to the VPS26-VPS29-VPS35 heterotrimer core that is found in mammals (VPS26 has two isoforms in mammals).

The retromer complex serves as a hub for recruiting accessory proteins and complexes, such as receptor-mediated endocytosis-8 (RME-8) (Freeman et al., 2014), Eps15 homology domain-containing protein-1 (EHD1) (Gokool et al., 2007), TBC1D5 (Seaman et al., 2009), and the WASH complex (Kvainickas et al., 2017; Simonetti et al., 2017), that regulate retromer’s role in endosomal trafficking and endosomal tubule dynamics (Harbour et al., 2010). For example, VPS35 binds to SNX3 and the GTPase Rab7, leading to membrane recruitment of retromer (Burd and Cullen, 2014; Harrison et al., 2014). While SNX3 directs retromer to early endosomes by binding to phosphatidylinositol 3-phosphate [PtdIns(3)P] (Harterink et al., 2011), Rab7-GTP recruits retromer to late endosomes (Rojas et al., 2008; Progida et al., 2010; Harrison et al., 2014). TBC1D5, which binds to VPS35 and VPS29, negatively regulates membrane recruitment of retromer by acting as a GTPase-activating protein for Rab7 (Seaman et al., 2009; Jia et al., 2016; Borg Distefano et al., 2018). Another important accessory protein is the WASH complex protein FAM21, which binds directly to VPS35 through its C-terminal repeats of the LFa motif (Gomez and Billadeau, 2009; Harbour et al., 2012; Jia et al., 2012; Helfer et al., 2013; Chen et al., 2019). WASH, which is regulated by MAGEL2 and USP7, then nucleates actin on the membrane (Hao et al., 2013; Hao et al., 2015; Florke Gee et al., 2020). The interaction between WASH and the Prader-Willi protein MAGEL2 will be discussed further in the last section.

### 5.2 Retromer and the sorting nexin protein family

Retromer functions as a coat complex that packages and delivers its cargo via tubular or vesicular structures to the TGN or plasma membrane (Lucas and Hierro, 2017; Wang et al., 2018). Vesicles coated with retromer are defined as retromer-coated endosomal tubular carriers (ETCs). Compared to other protein coats (i.e., clathrin, COPI, and COPII), ETCs are much more heterogeneous with a “loose” assembled coat, possibly an adaptation to different membrane curvatures (Chen et al., 2019). Retromer coat assembly depends on SNX proteins in mammals, as retromer does not possess intrinsic membrane-binding properties (Burd and Cullen, 2014; Chen et al., 2019). Different combinations of SNX proteins and retromer are important for recycling specific proteins (Gallon and Cullen, 2015; Chamberland and Ritter, 2017; Yong et al., 2022).

The SNX protein family expanded from 10 proteins in yeast to 33 in mammals and six of them (SNX1, SNX2, SNX3, SNX5, SNX6, and SNX27) were shown to associate with the retromer complex (Cullen, 2008; Burd and Cullen, 2014; Lucas and Hierro, 2017). SNX1 and SNX2 are Vps5 orthologs, and SNX5 and SNX6 are Vps17 orthologs (McGough and Cullen, 2011). All SNX proteins possess a Phox (PX) domain that binds to phospholipids, in particular PtdIns(3)P, found in endosomes (Bugarcic et al., 2011; Chamberland and Ritter, 2017; Wang et al., 2018). SNX proteins are divided into subfamilies based on their domain architecture (Wang et al., 2018). SNX-PX subfamily members, like SNX3, only possess a PX domain. The SNX-FERM (4.1/ezrin/radixin/moesin) subfamily member SNX27 contains a FERM domain, which can bind to PtdIns(3)P and NPxY motifs, and a PDZ domain, which facilitates protein-protein interactions (Zhang et al., 2018). The SNX-BAR subfamily members contain a BAR domain that is capable of sensing and inducing membrane curvature (Burd and Cullen, 2014). The retromer-interacting proteins from the SNX-BAR subfamily include SNX1, SNX2, SNX5, and SNX6 (Lucas and Hierro, 2017). Although most SNX proteins associate with early endosome-enriched PtdIns(3)P, some also interact with late endosome-enriched phosphatidylinositol 3,5-phosphate [PtdIns(3,5)P]. SNX-PX-retromer and SNX-BAR-retromer mediate retrograde transport to the TGN, and SNX27-retromer mediates recycling to the plasma membrane through its PDZ domain (Temkin et al., 2011; Steinberg et al., 2013; Lucas and Hierro, 2017). Besides binding phosphatidylinositides, SNXs also play a central role in cargo recognition (Wang et al., 2018).

Retromer cargo selection is facilitated directly by VPS35 and VPS26 and/or by adaptor proteins, like SNXs, AP-1, and GGAs (Burd and Cullen, 2014; Cullen and Steinberg, 2018). The cargo proteins CI-MPR, sortilin, Wntless, and polymeric immunoglobulin (pIg) receptor possess a ΦX(L/M/V), where Φ is F/Y/W, consensus sequence that facilitates direct binding to SNX3-retromer (Verges et al., 2004; Seaman, 2007; Canuel et al., 2008; Harterink et al., 2011; Harrison et al., 2014; Lucas and Hierro, 2017; Cui et al., 2019; Yong et al., 2022). In general, at least a hydrophobic (F/W)L(M/V) motif that is commonly present in cargo proteins is needed for retromer-mediated sorting (Seaman, 2007; Cullen and Steinberg, 2018). This direct retromer interaction pathway is likely conserved in all eukaryotes. In contrast, metazoan-specific adaptor-dependent sorting allowed for the evolution of a plethora of sorting signals to finetune retrograde trafficking (Cullen and Steinberg, 2018). For example, a bipartite motif in VPS10, the functional homolog of CI-MPR in yeast, is needed for precise recognition by yeast retromer subunits Vps26 and Vps35 but not Vps17 (Suzuki et al., 2019). However, mammalian SNX5/SNX6 (yeast orthologs of Vps17) may function as the cargo-selecting module that recognizes a bipartite motif in CI-MPR and many other cargo proteins (Kvainickas et al., 2017; Simonetti et al., 2017; Simonetti et al., 2019; Yong et al., 2020). As another example, metazoan-specific SNX27 acts as an adaptor protein to select cargo through its FERM and PDZ domains (Cullen and Steinberg, 2018; Chen et al., 2019). Examples of SNX27 cargo containing a PDZ-binding motif [i.e., (S/T)xΦ] include β2-adrenergic receptor, parathyroid hormone receptor 1 (PTHR), α-amino-3-hydroxy-5-methyl-4-isoxazolepropionic acid (AMPA) receptor, and the N-methyl-D-aspartate (NMDA) receptor, which are all important in neuroendocrine tissues (Temkin et al., 2011; Steinberg et al., 2013; Lucas and Hierro, 2017). Some of these proteins, particularly their sorting motifs, must be phosphorylated to facilitate high-affinity binding to SNX27, suggesting additional complexity in regulation and finetuning of retrograde transport (Clairfeuille et al., 2016; Lucas and Hierro, 2017).

### 5.3 Retromer, the WASH complex, and F-actin in retrograde trafficking

An essential part of endosomal recycling is membrane remodeling, which is mediated by patches of branched F-actin, and requires membrane localization of the retromer, WASH, and ARP2/3 complexes (Fokin and Gautreau, 2021). Endosomal cargo proteins are recognized by specific combinations of retromer and SNXs (Gallon and Cullen, 2015; Chamberland and Ritter, 2017; Yong et al., 2022). SNX proteins localize the retromer complex to the membrane, and FAM21 binds to retromer’s VPS35 to facilitate membrane localization of the WASH complex (Harbour et al., 2010; Bugarcic et al., 2011; Harbour et al., 2012; Jia et al., 2012; Helfer et al., 2013; Seaman and Freeman, 2014; Chamberland and Ritter, 2017). A recent study indicates that SWIP/KIAA1033 can also recruit the WASH complex to the endosomal membrane independently of the FAM21-VPS35 interaction (Dostal et al., 2023). The endosomal membrane recruitment of WASH complex may also be mediated through interaction with SNX27 (Temkin et al., 2011; Steinberg et al., 2013) or its own direct interaction with endosomal lipids (Derivery et al., 2009).

The WASH complex functions as the major actin nucleation-promoting factor in endosomal recycling and is required for the recycling of several proteins, including CI-MPR, glucose transporter 1 (GLUT1), α5β1 integrin, and major histocompatibility complex II (MHC II) (Gomez and Billadeau, 2009; Zech et al., 2011; Piotrowski et al., 2013; Graham et al., 2014; Dostal et al., 2023). WASH possesses a conserved C-terminal VCA (verprolin homologous or WASP-homology-2, connector, and acidic) domain that binds actin monomers and ARP2/3 to trigger actin filament nucleation (Linardopoulou et al., 2007; Duleh and Welch, 2010). More specifically, binding of ARP2/3 to WASH’s VCA domain induces a conformational change in ARP2/3 that loads the first actin monomer and allows further actin polymerization through rapid ATP hydrolysis on ARP2 (Derivery et al., 2009; Padrick et al., 2011; Espinoza-Sanchez et al., 2018). Prior to actin nucleation, WASH’s VCA domain is autoinhibited by intra- and intermolecular interactions (Hao et al., 2013). Small GTPases and PtdIns(4,5)P are some general regulators that may release VCA motif inhibition, but tissue-specific machinery, like the MUST complex (composed of MAGEL2, TRIM27 and USP7) might have evolved to enable better adaptation to specific environmental challenges (Figure 6) (Fon Tacer and Potts, 2017; Lee and Potts, 2017; Florke Gee et al., 2020).

The WASH complex promotes retromer trafficking by triggering actin polymerization via ARP2/3 complex recruitment and activation. Actin polymerization combined with the action of SNX-BAR proteins and motor proteins ultimately leads to the formation of tubular structures (Fokin and Gautreau, 2021). Subsequent actin polymerization and the activity of the dynein-dynactin complex leads to the fission of tubular vesicles that carry various cargo proteins to their final destinations (Hunt et al., 2013; Wang et al., 2018; Fokin et al., 2021). This pathway is well-established for diverse constitutively secreted receptors; however, recent evidence suggests that essential SG membrane proteins and granins are recycled back to the TGN for nascent granule formation by retromer-dependent retrograde transport, though the mechanistic details and sorting motifs are still more or less unknown (Ferraro et al., 2005; Ma et al., 2020; Neuman et al., 2021). Interestingly, Prader-Willi mouse models with depletion of *Magel2* suggested that, in the hypothalamus, SG-resident proteins are the major targets of Magel2-retromer-dependent protein recycling (Figure 6) (Fon Tacer and Potts, 2017; Lee and Potts, 2017; Florke Gee et al., 2020).

## 6 MAGEL2 in regulated secretion of the hypothalamus

MAGEL2 is a mammalian-specific member of the melanoma antigen gene (MAGE) family that is specifically expressed in the brain and highly enriched in the hypothalamus (Figure 3) (Hao et al., 2015; Fon Tacer et al., 2019). Like many MAGE family proteins, MAGEL2 functions as a regulator of an E3 ubiquitin ligase (Doyle et al., 2010; Lee and Potts, 2017; Florke Gee et al., 2020). In particular, MAGEL2 interacts with the RING E3 ligase TRIM27 and VPS35 in the retromer complex, thus facilitating the localization of TRIM27 to retromer-positive endosomes (Figure 6A) (Hao et al., 2013). MAGEL2-TRIM27 promotes K63-linked polyubiquitination of WASH K220 (Hao et al., 2013). Unlike some other ubiquitin linkage types, K63-linked ubiquitination generally acts as a signaling event rather than targeting a protein for proteasomal degradation. Accordingly, MAGEL2-TRIM27-mediated ubiquitination leads to WASH activation and actin polymerization (Hao et al., 2013; Hao et al., 2015; Fon Tacer and Potts, 2017). The deubiquitinase USP7 forms a complex with MAGEL2-TRIM27 and preferentially cleaves K63-linked ubiquitin chains (Hao et al., 2015). Thus, USP7 fine-tunes F-actin levels in the endosome by counteracting TRIM27 activity and preventing the overactivation of WASH (Hao et al., 2015). Although retromer is the major player in endosomal retrieval and recycling, other similar complexes also recycle proteins (McNally and Cullen, 2018). Interestingly, the WASH complex can also associate with commander/CCC/retriever complex (Phillips-Krawczak et al., 2015; McNally et al., 2017; Chen et al., 2019); however, the role of MAGEL2 in activating WASH in association with this complex is not known and warrants future investigation. Furthermore, why WASH needs finetuning by MAGEL2 in the hypothalamic neurons is the subject of current research.

The hypothalamus is a region of the brain that integrates signals from different sensory inputs to maintain homeostasis by reacting and adapting to any changes or stressors in the environment. By controlling the autonomic nervous system via neurons linking it to both the parasympathetic and sympathetic systems, the hypothalamus regulates body temperature, hormones (e.g., thyrotropin-releasing hormone, gonadotropin-releasing hormone, somatostatin, and dopamine), and behavioral responses. Hormones released from hypothalamic neurons travel through the hypophyseal portal system to the pituitary gland, controlling the release of other hormones that regulate various endocrine glands and organs. By linking the nervous and endocrine systems, this hypothalamus-pituitary axis acts as a major control center in the body (Nillni, 2007). Intriguingly, the diverse functions of the MAGE protein family are unified in their physiological function of stress adaptation (Fon Tacer et al., 2019; Florke Gee et al., 2020). *MAGEL2* is uniquely expressed in the hypothalamus, and the molecular understanding of its function suggests its role as a tissue-specific regulator of the retrograde recycling of SG components and neuroendocrine function in the hypothalamus (Hao et al., 2015; Fon Tacer and Potts, 2017).

Given that the hypothalamus plays a key role in regulating many physiological processes, perturbations in the regulated secretion pathway lead to improper hypothalamic development and function that phenotypically manifests in syndromes, such as PWS and Schaaf-Yang syndrome (SYS) (Hoyos Sanchez et al., 2023). PWS is a complex neurogenetic disorder caused by paternal loss of the maternally imprinted 15q11-q13 chromosomal region (called the Prader-Willi region) that contains small nucleolar RNA genes and six protein-coding genes (*MKRN3*, *NDN*, *NPAP1*, *SNURF-SNRPN*, and *MAGEL2*) (Butler et al., 2019; Alves and Franco, 2020). While the loss of *MAGEL2* affects regulated secretion in the hypothalamus (Figure 6B), loss of the other Prader-Willi region genes also impacts the clinical presentation of PWS (Cassidy and Driscoll, 2009; Fon Tacer and Potts, 2017; Chen et al., 2020). Currently, PWS and SYS have no cure or effective therapy, and treatment focuses on managing the symptoms that arise from perturbations in the regulated secretion pathway and the dysregulation of crucial hypothalamic hormones (Alves and Franco, 2020; Hoyos Sanchez et al., 2023). Given that PWS is relatively common and affects 1 in 15,000 children, there is a pressing need to find better treatment options (Cassidy and Driscoll, 2009). The recent findings about MAGEL2’s role in regulated secretion suggest that restoring this pathway may alleviate multiple symptoms of PWS and other similar neurodevelopmental disorders (Chen et al., 2020).

While the mechanisms of retrograde trafficking are extremely complex, cargo sorting at the endosomes culminates in ARP2/3 activation and, in turn, actin nucleation. Through facilitating the activation of WASH, MAGEL2 functions as a tissue-specific regulator of ARP2/3 activation in the hypothalamus (Hao et al., 2013; Hao et al., 2015). The loss of *MAGEL2* expression causes deficits in SG abundance and bioactive neuropeptide production, impacting hypothalamic neuroendocrine function and contributing to the clinical presentation of PWS (Figure 6B) (Chen et al., 2020; Hoyos Sanchez et al., 2023). In particular, reduced levels of the neuropeptides vasopressin, galanin, oxytocin, proenkephalin, somatostatin, and thyrotropin-releasing hormone and the SG components involved in their processing and release (i.e., CHGB, secretogranin II and III, PC1/3, PC2, and CPE) have been discovered in PWS mouse models and patient-derived neuronal cell models (Chen et al., 2020; Hoyos Sanchez et al., 2023).

## 7 Conclusion

Together, several decades of research provided important insights into the complexity of vesicle trafficking and protein sorting in neuroendocrine cells. In the regulated secretory pathway, the anterograde transport is compensated by the retrograde movement of lipids and proteins to maintain membrane homeostasis and recycle various proteins. Intriguingly, the recycling of constitutive proteins of secretory granules (e.g., PCs, CPE, and granins) that enable hormone and neuropeptide maturation and cargo condensation, emerged as critical for proper hypothalamic secretion. Furthermore, actin polymerization at the site of protein sorting and vesicle budding controls retrograde transport and is regulated in a tissue-specific manner by WASH complex and MAGEL2 in the hypothalamus. Intriguingly, the diverse functions of the MAGE protein family are unified in their physiological function of stress adaptation. Given that *MAGEL2* is uniquely expressed in the hypothalamus, the molecular understanding of its function suggests its role to enable better and faster adaptation to an ever-changing environment. The loss of *MAGEL2* ultimately leads to insufficient F-actin nucleation and a decreased percentage of SG proteins recycled, which manifests in a perturbation of the hormonal secretion in patients with Prader-Willi and Schaaf-Yang syndromes. Further uncovering of the tissue-specific regulation of anterograde and retrograde transport thus offers potential therapeutic opportunities for patients.

## Abbreviations

SG, secretory granule; ISG, immature secretory granule; SeV, secretory vesicle; SV, synaptic vesicle; PC, proprotein convertase; CPE, carboxypeptidase E; M6P, mannose-6-phosphate; MPR, mannose-6-phosphate receptor; RSPs, regulated secretory proteins; AP-1, adaptor protein 1; GGA, Golgi-localized, γ-ear containing, ARF-binding; CKII, casein kinase II; PWS, Prader-Willi syndrome; SYS, Schaaf-Yang syndrome.
